# A Metadata Analysis of Oxidative Stress Etiology in Preclinical Amyotrophic Lateral Sclerosis: Benefits of Antioxidant Therapy

**DOI:** 10.3389/fnins.2018.00010

**Published:** 2018-01-24

**Authors:** Leila Bond, Kamren Bernhardt, Priyank Madria, Katherine Sorrentino, Hailee Scelsi, Cassie S. Mitchell

**Affiliations:** ^1^Laboratory for Pathology Dynamics, Department of Biomedical Engineering, Georgia Institute of Technology and Emory University School of Medicine, Atlanta, GA, United States; ^2^Department of Chemistry and Biochemistry, Georgia Institute of Technology, Atlanta, GA, United States

**Keywords:** HSP70, oxidants, glutamate, rotarod, motoneuron disease, antioxidants, vitamins

## Abstract

Oxidative stress, induced by an imbalance of free radicals, incites neurodegeneration in Amyotrophic Lateral Sclerosis (ALS). In fact, a mutation in antioxidant enzyme superoxide dismutase 1 (SOD1) accounts for 20% of familial ALS cases. However, the variance among individual studies examining ALS oxidative stress clouds corresponding conclusions. Therefore, we construct a comprehensive, temporal view of oxidative stress and corresponding antioxidant therapy in preclinical ALS by mining published quantitative experimental data and performing metadata analysis of 41 studies. *In vitro* aggregate analysis of innate oxidative stress inducers, glutamate and hydrogen peroxide, revealed 70–90% of cell death coincides to inducer exposure equivalent to 30–50% peak concentration (*p* < 0.05). A correlative plateau in cell death suggests oxidative stress impact is greatest in early-stage neurodegeneration. *In vivo* SOD1-G93A transgenic ALS mouse aggregate analysis of heat shock proteins (HSPs) revealed HSP levels are 30% lower in muscle than spine (*p* < 0.1). Overall spine HSP levels, including HSP70, are mildly upregulated in SOD1-G93A mice compared to wild type, but not significantly (*p* > 0.05). Thus, innate HSP compensatory responses to oxidative stress are simply insufficient, a result supportive of homeostatic system instability as central to ALS etiology. *In vivo* aggregate analysis of antioxidant therapy finds SOD1-G93A ALS mouse survival duration significantly increases by 11.2% (*p* << 0.001) but insignificantly decreases onset age by 2%. Thus, the aggregate antioxidant treatment effect on survival in preclinical ALS is not sufficient to overcome clinical heterogeneity, which explains the literature disparity between preclinical and clinical antioxidant survival benefit. The aggregate effect sizes on preclinical ALS survival and onset illustrate that present antioxidants, alone, are not sufficient to halt ALS, which underscores its multi-factorial nature. Nonetheless, antioxidant-treated SOD1-G93A ALS mice have significantly increased motor performance (*p* < 0.05) measured via rotarod. With a colossal aggregate preclinical effect size average of 59.6%, antioxidants are promising for increasing function/quality of life in clinical ALS patients, a premise worth exploration via low-risk nutritional supplements. Finally, more direct, quantitative measures of oxidative stress, antioxidant levels and bioavailability are key to developing powerful antioxidant therapeutics that can assert measurable impacts on redox homeostasis in the brain and spinal cord.

## Introduction

Amyotrophic lateral sclerosis (ALS) is a fatal neurodegenerative disease characterized by the selective loss of motor neurons throughout the nervous system (Irvin et al., 2015). Patients experience progressive muscular weakness, dysphagia, and respiratory dysfunction that usually leads to death within about 3 years of symptom onset (Coan and Mitchell, 2015). So far, only two United States Food and Drug Administration (FDA) approved therapies exist, neither of which are capable of curing ALS. The multi-factorial and multi-scalar nature of ALS (Mitchell and Lee, 2012b; Irvin et al., 2015; Kim et al., 2016) has made etiological and therapeutic exploration difficult. As such, the precise impact of oxidative stress, one of the first identified contributors to ALS neurodegeneration, has remained elusive.

Oxidative stress exacerbates homeostatic dysregulation of mitochondria, proteins, genes, and other cellular processes. Extensive research continues to be conducted on ALS pathophysiology, including oxidative stress, in transgenic ALS mouse models. The superoxide disumutase-1 glycine 93 to alanine (SOD1-G93A) transgenic mouse is the most common preclinical model for these kinds of research projects (Pfohl et al., 2015). Correspondingly, human SOD1 variants are some of the most common mutations in clinical familial ALS (Petri et al., 2006a). The high copy SOD1-G93A B6SJL transgenic mouse reliably produces an ALS phenotype with an average onset and lifespan of 99 and 130 days, respectively, (Pfohl et al., 2015).

SOD1 is an antioxidant enzyme that decomposes harmful superoxide radicals into molecular oxygen (O_2_) and hydrogen peroxide (H_2_O_2_), which are less harmful to cells (Pehar et al., 2014). H_2_O_2_, in and of itself, is still an oxidant, especially at higher concentrations, and is further broken down by another enzyme called catalase. SOD1 mutations result in impaired cellular metabolism and respiration (Richardson et al., 2013), and the insurmountable build-up of misfolded mutant SOD1 protein aggregates (Kim et al., 2016). In addition to neutralizing damaging oxidants, naturally occurring “normal” SOD1 in the spinal cord also has anti-apoptotic properties due its localization and interaction in the outer mitochondrial membrane with BCL-2 (Rosen et al., 1993).

Due to the presence of SOD1 mutations, SOD1-G93A transgenic ALS mice experience pronounced increases in oxidative stress, which is hypothesized to significantly contribute to ALS-related motoneuron degeneration due to the effects of oxidative stress and secondary effects in numerous downstream pathways (Richardson et al., 2013). For example, neurons have voltage-gated calcium channels in their lipid bilayer membranes, and the reactive oxygen species (ROS) that are responsible for oxidative stress cause a conformational change in the channel proteins that allows calcium influx. The positive charge from the Ca^2+^ depolarizes the mitochondria, decreasing their ability to carry out respiration (Andreassen et al., 2001a) and leading to apoptosis. In fact, our lab's prior work showed that mitochondrial respiration is decreased throughout the entire lifespan of SOD1-G93A ALS mice (Irvin et al., 2015). The higher concentration of ROS also causes protein misfolding, further exacerbating protein dysregulation and transport deficiencies that strangle the cell (Mitchell and Lee, 2012a; Kim et al., 2016). Furthermore, oxidative stress leads to a reduction in glial cells (Park et al., 2007), which decreases efficacy of neuron firing due to axonal signal dissipation. Innate heat shock proteins like HSP70 are thought to be the body's primary compensatory mechanism countering oxidative stress (Wei et al., 2013), although there are also endogenous antioxidants, like glutathione (Irvin et al., 2015).

While several experimental studies have examined preclinical oxidative stress, individual study sample sizes have limited their conclusions' strength. The goal of this study is to gain a more holistic view of oxidative stress in ALS and its importance as an ALS therapeutic target. We perform a metadata analysis, which aggregates *In vitro* and *in vivo* data across numerous SOD1-G93A ALS mouse studies to create a much larger sample size, enabling overarching conclusions on the etiology of oxidative stress and innate compensatory HSPs. Assessed outcome metrics include correlations of oxidative stress with *In vitro* cell viability, *in vivo* correlation to ALS muscle function decline, and ALS mouse onset age and survival duration. Using this comprehensive perspective, we assess prioritization of antioxidant therapeutic development in clinical ALS to prolong survival and increase patient quality of life.

## Methods

We perform a metadata analysis to construct a comprehensive view of oxidative stress in ALS using published experimental data to perform aggregate statistical analysis with sample sizes that enable stronger conclusions. The general method involved (1) mining, selecting and recapturing published data from preclinical ALS experiments examining or treating oxidative stress; (2) normalizing recaptured data to enable aggregation across studies; (3) analyzing aggregate data using appropriate statistical methods.

### Data source identification and inclusion

Keywords were used to identify potential data sources in PubMed/Medline. All potential data sources were initially searched using key words “Amyotrophic Lateral Sclerosis” OR “ALS” AND “transgenic mouse.” Searches were limited to articles published in English and with publication dates through year-2016. Primary search articles were downloaded into a Filemaker Pro relational database (Mitchell et al., 2015a; Kim et al., 2016). Secondary searches within the Filemaker Pro database were performed using “ox*” (to represent all words related to oxidation, oxidative stress, or antioxidants) OR “heat shock proteins,” including all synonyms. A script was written for FileMaker Pro to isolate papers that had any mention of oxidative stress, oxidants, antioxidants, or heat shock proteins. Upon applying the detailed study-specific inclusion criteria outlined below, a total of 41 papers with quantifiable experimental data were ultimately included for analysis.

#### *In vitro* oxidative stress data sources

*In vitro* analysis of oxidative stress utilized experiments performed within isolated cell cultures. Included data sources measured oxidant concentration and cell viability on nonspecific murine derived cell lines, including wild type and SOD1-G93A cells.

#### *In vivo* heat-shock proteins data sources

Heat shock protein (HSP) analysis consisted of *in vivo* experiments assessing HSP response as a function of time and/or disease progression. Included data measured concentration of *any* HSP in tissue samples from wild-type and SOD1-G93A mice (B6SJL or C57BL/6 strains). Data was included, but analyzed separately, for two different anatomical locations: spinal cord tissue and muscle tissue.

#### *In vivo* oxidative stress data sources

*In vivo* analysis assessed the impact of oxidative stress and therapies that target oxidative stress. Included data sources quantitatively measured mouse survival, ALS onset age, or mouse rotarod performance. Due to known significant differences in ALS onset age, ALS mouse data sources were restricted to the more common SOD1-G93A B6SJL strain (Pfohl et al., 2015). Wild type data from each data source was extracted for comparison. Data from treatments intended to target oxidative stress, such as antioxidants or substances that implicitly increase antioxidant levels, were included; treatments that intentionally modified SOD1-G93A B6SJL or wild type mouse genetics or utilized other forms of genetic engineering were excluded.

### Data recapture

Data recapture was performed as previously described (Mitchell et al., 2015a). Briefly, articles were downloaded from either PubMED Central or from e-journal subscriptions available through the Georgia Institute of Technology and Emory University libraries. Recaptured data was transcribed from articles into a relational Filemaker Pro database (Kim et al., 2016). Quantitative data was transcribed from individual articles and independently assessed for accuracy by a quality control team using previously published biocuration protocols (Mitchell et al., 2015a) to insure transcription accuracy.

### Data aggregation and normalization

Quantitative data was aggregated and normalized on a sub-study basis: *In vitro* analysis oxidative stress, *in vivo* analysis of heat shock proteins, and *in vivo* analysis of oxidative stress and anti-oxidant therapies.

#### *In vitro* oxidative stress measurements

“Treated” *In vitro* oxidative stress data was normalized to paired non-treated control data from each independent data source. To compensate for varying terminology among articles, “cell death” was used as an overarching term encompassing three related, inter-dependent measures: cell viability, cell survival, and cell death. Cell survival and cell viability measures were converted into cell death by subtracting the probability from 1.

#### *In vivo* heat shock protein measurements

To account for the variation of heat shock protein (HSP) level measurement methods between articles, HSP level data were normalized by calculating the ratios of transgenic to WT HSP levels, (G93A HSP/WT HSP). Data from each study were normalized to their respective WT data, and ratios obtained from each article were weighted equally using reported sample size (number of mice). HSP data in the “limbs” characterization include data from articles that used a combination of the soleus, transverse abdominal (TA), and extensor digitorum longus (EDL) muscles from the hind limbs of B6SJL or C57BL/6 mice. The overall HSP analysis included all HSP types. The HSP70 analysis explicitly included measures of HSP70, which is the most commonly measured HSP.

#### *In vivo* assessment of antioxidants, functional outcome measurements

*In vivo* SOD1-G93A transgenic ALS mouse data was aggregated into control and treatment groups to independently assess the impact on three functional outcome measurements: ALS onset age, survival, and rotarod performance. Treatment groups included all treatments meant to counteract oxidative stress. Antioxidant treatments varied, but examples include AEOL 10150 (Petri et al., 2006a), metalloporphyrins such as iron porphyrins (Wu et al., 2003; Kiaei et al., 2005), EGb761 (Ferrante et al., 2001). All recaptured data points were utilized without subsequent post-processing with one exception—the treatment start day analysis, which compared the impact of antioxidant treatment start time on functional outcome. For the latter case, survival values were normalized to the untreated control(s).

### Statistical analysis

Multiple analyses in this study (i.e., *In vitro* oxidative stress, *in vivo* heat shock proteins, and portions of the *in vivo* functional outcomes assessment) involved the comparison of averages between treatment and control groups. Prior to selection of a statistical analysis method, data distribution type was determined using a Shapiro-Wilk test. Subsequently, the appropriate test, either a one-tailed two-sample *t*-test or a Mann-Whitney U-test, was implemented in Stata (StataCorp, USA) and Matlab (The Mathworks, Inc.). Alpha selection for individual analyses are as stated in the Results.

When analyzing temporal rotarod performance data, ANOVA and paired *T*-tests were performed on rotarod times (in seconds) for the treated mice vs. all of the rotarod times of the untreated mice of the same age (in days). To adjust for multiple comparisons, a Bonferroni correction was used to lower the *p*-value significance threshold to *p* < 0.025. Similarly, paired *T*-tests with a Bonferroni Correction of *p* < 0.0125 were also performed on the treatment vs. control data points in each of the individual rotarod time bins that compared changes in rotarod performance with age [analogous to ALS disease progression].

To assess the impact of antioxidant treatment start date on ALS survival, a histogram was first made to determine the distribution of *in vivo* SOD1-G93A data by treatment start date. Based on the overall distribution, two distinct pools of data were identified: “early” pre-onset treatments starting before post-natal day 42 and “late” pre-onset treatment starting after post-natal day 42. The centers of these two corresponding bins were approximately 30-days and 60-days, respectively. Day 42, itself, was removed from the analysis. Note that there is no particular significance of day 42, itself, other than it happens to be the natural bi-modal cut-off for the distribution of treatment start dates obtained from the included studies. Mann Whitney U-test with significance set for *p* < 0.05 was conducted to assess differences between the average survival durations of the early and late pre-onset antioxidant treatment groups. Analysis of post-onset antioxidant treatment initiation was not possible due to a lack of experimental data (see Unraveling the Impact and Timing of Preclinical Antioxidant Therapy).

## Results

The presented composite, temporal assessment of oxidative stress in preclinical ALS had three key components. First, the aggregate impact of oxidative stress was analyzed *In vitro* to assess the trends of specific cytotoxic oxidative stress inducers on cell death. Next, *in vivo* heat shock proteins were examined in aggregate to quantify the response magnitude of innate biological mechanisms meant to compensate for oxidative stress. Finally, *in vivo* antioxidant treatments were analyzed to determine their aggregate, average impact on temporal functional disease measurements, including muscle function assessed via rotarod, ALS symptom onset age, and time of death (or ALS disease duration). A total of 41 peer-reviewed, published scientific articles met the study inclusion and exclusion criteria. Data categorization and breakdown can be found in Table 1.

**Table 1:** Data categorization, samples sizes, and sources.

**Category**	**Sub-category**	**Articles**	**Sample Size**	**References**
***In vitro***	All	8	47	Kriscenski-Perry et al., 2002; Mahoney et al., 2004; Petri et al., 2006b; Park et al., 2007; Sharp et al., 2008; Moges et al., 2009; Lucchetti et al., 2013; Richardson et al., 2013.
	Glutamate	4	18	Kriscenski-Perry et al., 2002; Mahoney et al., 2004; Sharp et al., 2008; Lucchetti et al., 2013.
	H_2_O_2_	3	15	Kriscenski-Perry et al., 2002; Mahoney et al., 2004; Moges et al., 2009.
	Paraquat	3	14	Petri et al., 2006b; Sharp et al., 2008; Richardson et al., 2013.
**HSP**	All	11	57	Kiaei et al., 2005; WHO, 2006; Yamashita et al., 2007; Kabashi et al., 2008; Kalmar et al., 2008; Kamat et al., 2008; Dadon-Nachum et al., 2011; Shin et al., 2012; Crippa et al., 2013; Wei et al., 2013; Mitchell et al., 2015a.
	Spine	5	41	Kiaei et al., 2005; WHO, 2006; Yamashita et al., 2007; Kalmar et al., 2008; Dadon-Nachum et al., 2011; Crippa et al., 2013; Wei et al., 2013; Mitchell et al., 2015a
	Muscle	6	16	Kiaei et al., 2005; WHO, 2006; Kabashi et al., 2008; Kamat et al., 2008; Shin et al., 2012; Crippa et al., 2013.
	HSP70	8	25	Kiaei et al., 2005; WHO, 2006; Yamashita et al., 2007; Kabashi et al., 2008; Kalmar et al., 2008; Kamat et al., 2008; Wei et al., 2013.
***In vivo***	All	22	187	Barneoud and Curet, 1999; Andreassen et al., 2001a,b; Ferrante et al., 2001; Kriscenski-Perry et al., 2002; Vleminckx et al., 2002; Klivenyi et al., 2004; Crow et al., 2005; Petri et al., 2006a; Xu et al., 2006; Shin et al., 2007; Yamashita et al., 2007; Orrell et al., 2008; Moges et al., 2009; Sekiya et al., 2009; Chen et al., 2010; Turner et al., 2010; Kong et al., 2012; Mimoto et al., 2012; Richardson et al., 2013; Miquel et al., 2014; Song and Chen, 2014
	Onset	8	16	Kriscenski-Perry et al., 2002; Vleminckx et al., 2002; Klivenyi et al., 2004; Petri et al., 2006a; Shin et al., 2007; Yamashita et al., 2007; Richardson et al., 2013; Song and Chen, 2014
	Survival	16	32	Andreassen et al., 2001a,b; Ferrante et al., 2001; Kriscenski-Perry et al., 2002; Vleminckx et al., 2002; Klivenyi et al., 2004; Crow et al., 2005; Petri et al., 2006a; Xu et al., 2006; Shin et al., 2007; Yamashita et al., 2007; Moges et al., 2009; Sekiya et al., 2009; Chen et al., 2010; Richardson et al., 2013; Song and Chen, 2014
	Rotarod	15	139	Barneoud and Curet, 1999; Andreassen et al., 2001a,b; Ferrante et al., 2001; Klivenyi et al., 2004; Petri et al., 2006a; Xu et al., 2006; Shin et al., 2007 Orrell et al., 2008; Sekiya et al., 2009; Turner et al., 2010; Kong et al., 2012; Mimoto et al., 2012; Richardson et al., 2013; Miquel et al., 2014

*The category states the type of data analyzed based on the sub-study definitions (see Methods), sub-category lists the individual metric(s) assessed, articles represents the number of included data source studies, sample size represents the number of included data points (cell cultures or mice), and references list the article citations for the included original data sources*.

### *In vitro* assessment of oxidative stress mechanisms

**Figure 1:**
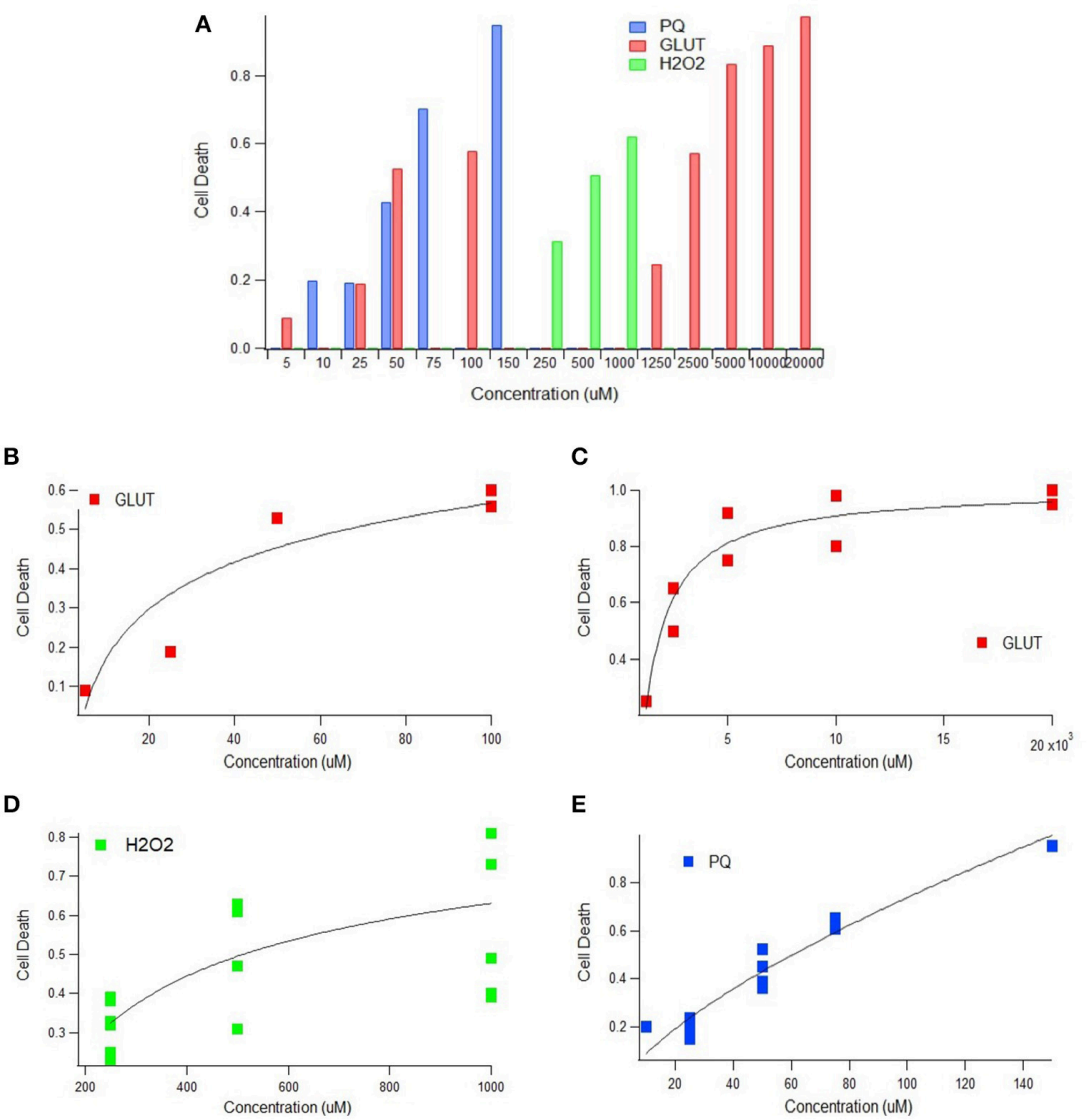
Cell death as a function of oxidant concentration *In vitro*. **(A)** *In vitro* cell death following exposure to paraquat, glutamate, or hydrogen peroxide concentrations (uM) (*p* < 0.05). **(B)** *In vitro* cell death trend plateaus following exposure to physiologically relevant glutamate concentrations (*p* < 0.05). **(C)** *In vitro* cell death trend following exposure of glutamate in excess of physiologically relevant concentrations (*p* < 0.05). **(D)** A scatter plot of concentration vs. cell death of 250, 500, and 1,000 uM of hydrogen peroxide (H_2_O_2_) (*p* < 0.05). Incubation times 2, 4, 6, and 10 h represented from bottom to top (*p* < 0.05). **(E)** *In vitro* cell death trend following exposure to paraquat, an extrinsic oxidative stress inducer (*p* < 0.05).

Multiple physiological (e.g., intrinsic) and non-physiological (e.g., extrinsic) oxidative inducers were assessed in order to determine the quantitative relationship between oxidative inducer concentration and cell death. Cell death was plotted against the concentration of glutamate (intrinsic inducer), hydrogen peroxide (intrinsic inducer), and paraquat (extrinsic inducer); in all instances, cell death increased significantly with oxidative inducer concentration (Figure 1A). Thus, both intrinsic and extrinsic oxidants are harmful to cells.

#### Impact of intrinsic oxidative inducers on cell death

Glutamate and hydrogen peroxide, two naturally occurring biological stressors known to induce oxidative stress, exhibited similar trends—a rapid increase in cell death at lower concentrations and then a plateau in cell death at higher concentrations. Trends are shown for glutamate for both “low,” or physiologically relevant concentrations (Figure 1B), and “high” glutamate concentrations, typically in excess of concentrations physiologically expected (Figure 1C). Both the low and high glutamate concentrations show a plateauing trend with cell death. The aggregated hydrogen peroxide data had two variables that were manipulated—oxidant concentration and incubation time. Cell death vs. concentration is shown with distinguishable trends for each incubation time (Figure 1D).

#### Impact of extrinsic oxidative inducers on cell death

In order to determine the extent of the plateau effect of oxidative stress inducers, a non-physiological or extrinsic stressor, paraquat, was examined to assess “extreme” cytotoxic conditions. However, the trend line shape of cell death vs. oxidative inducer concentration for the extrinsic paraquat was very different from that of the intrinsic inducers, glutamate and hydrogen peroxide. Paraquat, an oxidizing agent found in herbicides, shows a linear relationship between cell death and concentration (Figure 1E). Thus, unlike the intrinsic oxidative inducers, cell death does not appear to plateau with increased extrinsic inducer concentration.

### Regulatory compensation with heat-shock proteins in SOD1-G93A mice

Heat shock proteins (HSPs) are one of the primary innate mechanisms to protect against and/or compensate for oxidative stress. Aggregated HSP levels were assessed in spine and muscle tissue from SOD1-G93A from either the B6SJL or C57BL/6 mouse strain to determine the magnitude of compensatory response to ALS-associated oxidative stress. Given that the differences between the two SOD1-G93A strains appear to predominantly affect only functional outcome measurements (Pfohl et al., 2015), data from both strains were used to increase the sample size of the HSP analysis.

#### Elevation of SOD1-G93A HSP70 in the spine and muscles

**Figure 2:**
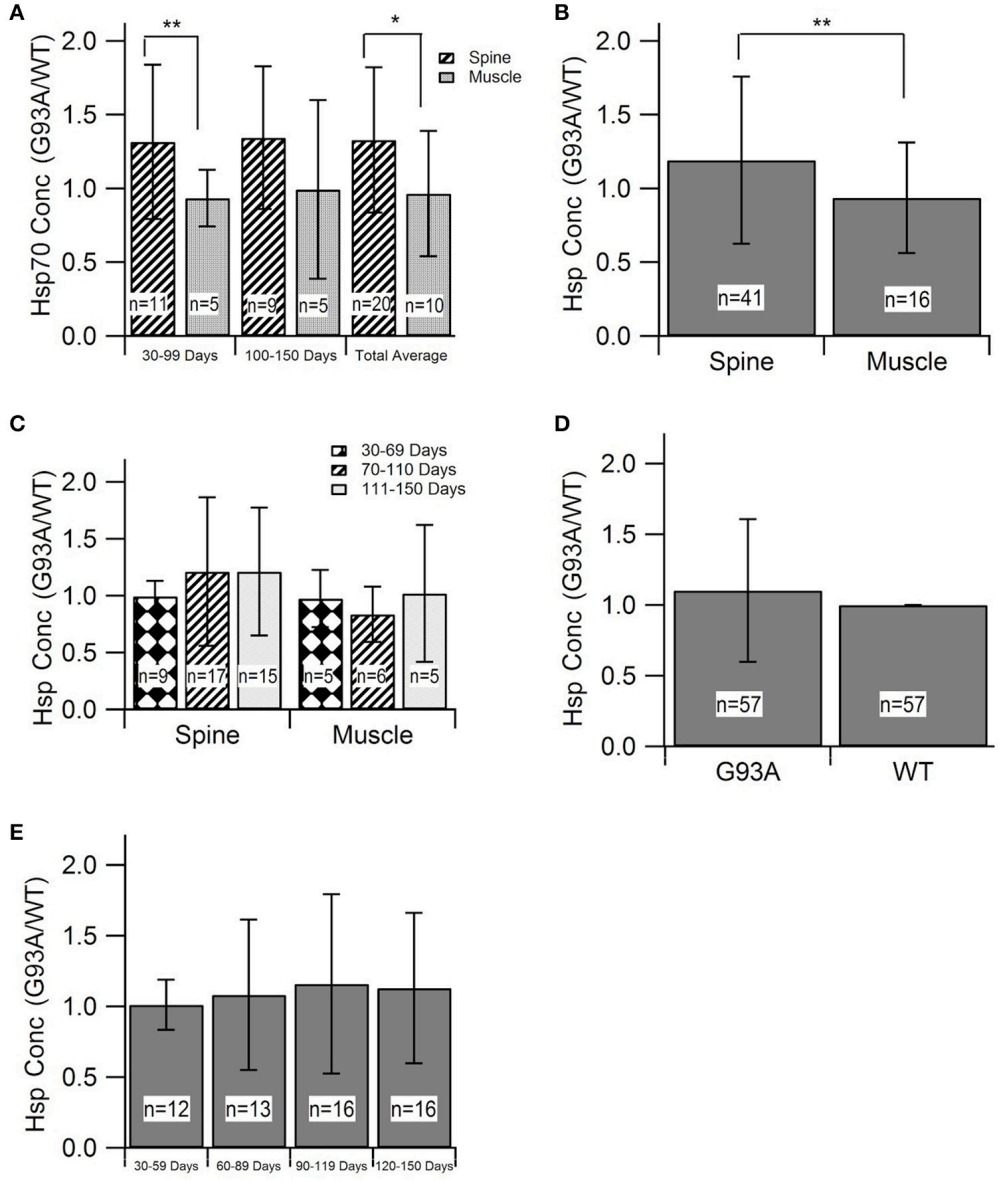
Heat-Shock Protein (HSP) concentration during disease progression in spine and muscle tissue samples from SOD1-G93A mice. Mann-Whitney U-Tests were used to determine any significance in all figure parts. **(A)** The G93A/WT ratio of HSP70 in the spine and muscle for three age groups. **(B)** The aggregate G93A/WT ratio of HSP70 concentration in the spine and muscle. **(C)** The G93A/WT ratio of overall HSP levels in the spine and muscle for three age groups. **(D)** The aggregate G93A/WT ratio of overall HSP concentration in the spine and muscle. **(E)** The G93A/WT ratio of overall HSP concentration for all locations combined and aggregated by age group. ^**^p < 0.05, ^*^p < 0.10.

Evaluating HSP levels in the spine and muscles is critical to determine potential inefficiencies of the heat-shock response in spinal motor neurons and muscle cells. HSP70 was the most prevalent HSP among the identified and included studies (8 of 11 articles and 30 of 57 data points). Due to sample size, HSP70 was the only individual HSP separately assessed in this aggregate metadata analysis. HSP70 data was divided into early (30–99 days) and late (100–150 days) stages of disease progression. The “early” group roughly aligns with visual symptom pre-onset and the “late” group roughly aligns with visual symptom post-onset. Average differences in HSP expression between spinal and muscle tissue samples was analyzed (Figure 2A). Results revealed by Mann-Whitney U-Test that, at an alpha value of 0.10, spinal HSP70 levels in the early stages of disease progression are significantly higher than muscle levels (*p* = 0.08). No significance was observed between early and late stage HSP70 levels within spinal or muscle data. The early and late stage data was then combined and the average HSP70 levels in spinal tissue samples were found to be significantly higher than the average HSP70 levels in muscle tissue samples.

#### Overall SOD1-G93A HSP trends in the spine and muscles

For assessment of overall trends in HSP levels in the spine and muscles, data from individual HSPs was combined into a single, overarching or aggregate group. Analysis of this data indicated that, at an alpha value of 0.10, HSP expression is significantly elevated in the spine compared to the muscles of SOD1-G93A mice (*p* = 0.0803) (Figure 2B). HSP levels in each tissue sample location were assessed at individual mouse age groups of 30–69, 70–110, and 111–150 days. These age groups, which were chosen based on statistical data distribution, roughly correlate with the functional asymptomatic disease stage; pre-onset to early symptom onset; and post-onset to end stage. Results generally indicated spinal tissue samples have consistent, qualitatively upregulated HSP levels, which are higher than the corresponding levels in muscle samples of the same age, albeit without statistical significance (Figure 2C). Of note is that there was one bin (days 30–69) in which there was no difference in HSPs between the spine and muscle.

#### Differences in SOD1-G93A HSP levels compared to wild type mice

To determine the overall effects of the SOD1-G93A ALS pathology on HSP concentration, the spinal and muscle tissue groups were combined into the “G93A” data group and compared to wild type “WT” data. As all HSP data was aggregated by finding the ratio of G93A to WT values, the value of WT data for analysis purposes was set to 1. To ensure equal weight of the two data sets, the sample size of the WT data was set equal to the sample size of the G93A data (*n* = 57). This data was first analyzed as an average value of all data points (Figure 2D). Average G93A HSP levels were upregulated compared to WT. However, Mann Whitney U-Test revealed no significance in this visually apparent increase in HSPs. G93A data was then separated into individual time bins of 30–59, 60–89, 90–119, and 120–150 days and compared to the WT average of 1 at each time bin (Figure 2E). In all cases, SOD1-G93A HSP levels were qualitatively upregulated compared to wild type, but again, no statistical significance was observed.

### *In vivo* assessment of preclinical SOD1-G93A antioxidant treatments

Thus far, aggregate analysis has focused on the mechanistic aspects of oxidative stress and HSP compensation for oxidative stress in ALS disease progression. However, in order to gain insight on the manifestations on functional disease outcomes, an *in vivo* comparison was conducted between SOD1-G93A (B6SJL strain) ALS control and “treated” mice. Aggregate outcome metrics included ALS onset age (when functional ALS symptoms first present), rotarod performance (the degree to which functional muscle symptoms progress during the disease), and survival duration (how long mice live after functional ALS symptom onset).

#### Effect of antioxidant treatment on ALS onset age

**Figure 3:**
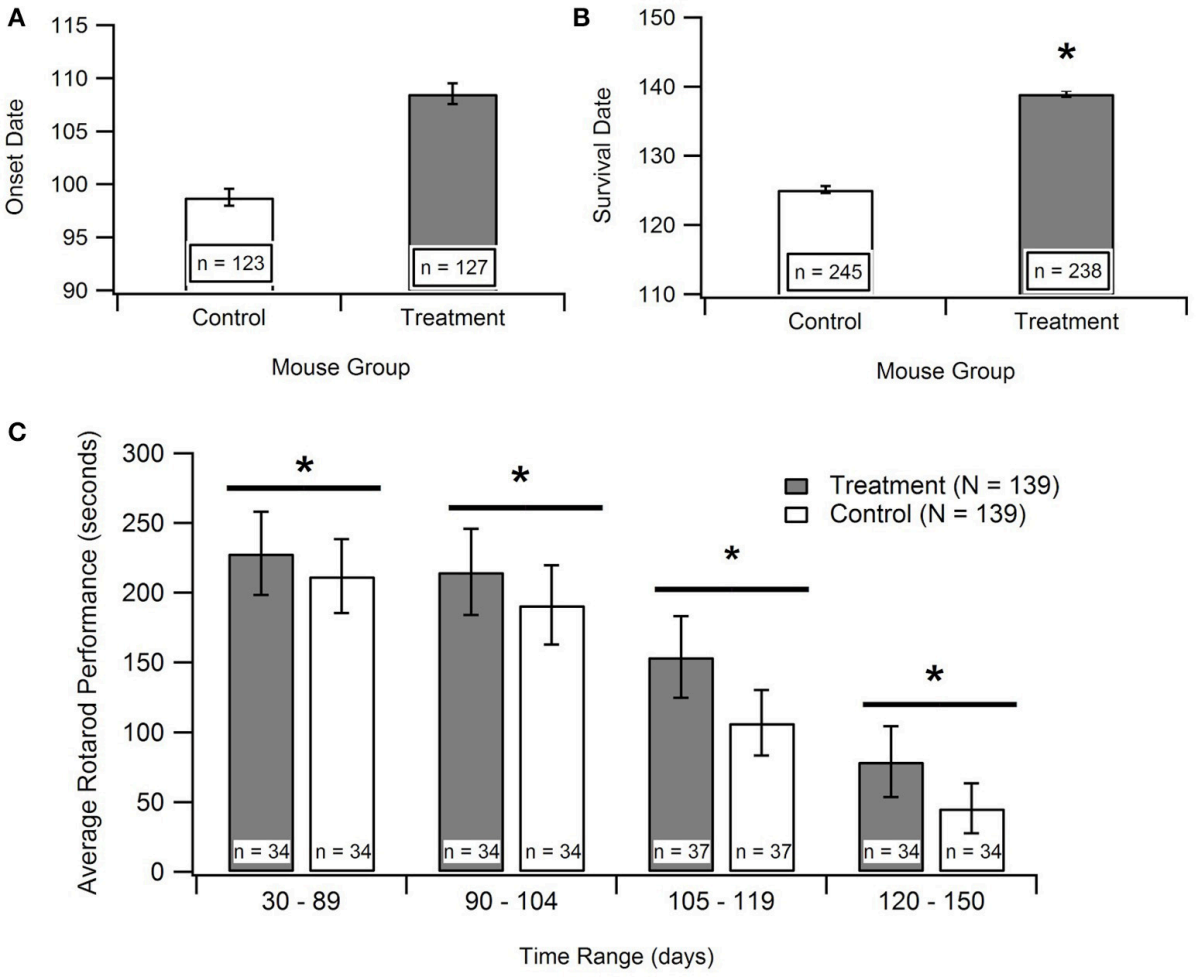
Effect of antioxidant treatment on *in vivo* outcome measures. Treatment group included SOD1-G93A mice given treatments to protect against oxidative stress. Control group included untreated SOD1-G93A mice. ^*^p < 0.05. **(A)** A Mann-Whitney U-test found no statistically significant difference between the mean onset dates of the control compared to the treatment (*p* > 0.05; *n* = number of mice). **(B)** A Mann Whitney U-test showed significant difference between survival dates of the treatment group vs. the control (*p* < 0.05). **(C)** Aggregated rotarod performance for SOD1-G93A mice treated with antioxidants and untreated SOD1-G93A mice (*n* = 139 total data points for each). ANOVA for overall treatment vs. control data (*p* < 0.05), *t*-tests for overall treatment vs. control (*p* < 0.05), and *t*-tests for treatment vs. control in each time range (in days), (*p* < 0.05). There is a consistent, significant difference between treatment and control rotarod performance.

The disease onset age for ALS SOD1-G93A transgenic mice treated with antioxidants [prior to their functional ALS disease onset] was assessed to determine if antioxidant therapy could delay ALS symptom onset. Figure 3A displays the aggregated data, which includes 16 data points encompassing 250 mice from 8 published studies. Subsequent statistical analysis of the aggregated data utilizing a Mann-Whitney U-test showed that antioxidant treatments do not significantly prolong onset date (*p* = 0.066). However, the *p*-value indicates a potential qualitative trend for treatments prolonging the onset of symptom. Sample size was a limiting factor of this analysis, and the inclusion of more studies could solidify the trend. While treating with antioxidants prior to symptom onset is not practical for clinical ALS, determining if antioxidants can delay ALS symptom onset in transgenic mice is still valuable for determining the degree and timing of oxidative stress contributions to the overall pathology.

#### Effect of antioxidant therapy on ALS motor performance

In order to examine the impact of antioxidant treatments during the survival interval, data was compiled for rotarod times of treated and untreated SOD1-G93A transgenic ALS mice from 15 separate studies. Rotarod is the industry standard for measuring mouse muscle function decline associated with ALS. The rod spins at a set rotational speed and the mice are timed to see how long they can stay on it without falling off. The rotarod tests both their muscular strength, namely paw grip strength, as well as muscular coordination. The published experimental studies utilized various types of antioxidant treatments, and each measured the rotarod performance of the treated and untreated SOD1-G93A mice throughout their life span. As shown in Figure 3C, the treatment group mice consistently stayed on the rotarod longer than control group mice of the same age. To determine if this difference was significant, an ANOVA and a *t*-test were performed on all the treatment data vs. all the control data. In addition, *t*-tests were performed on treatment vs. control data in each of the mouse age groups shown in Figure 3C. All of the tests had *p* < 0.05, and the comparable *p*-values (for the overall ANOVA and *t*-test, and for the individual age group *t*-tests) also passed the Bonferroni correction. Thus, the differences between the rotarod performance of control and treated mice were statistically significant both in the overall aggregate analysis as well as within each age group.

#### Effect of antioxidant therapy on ALS survival

Time of death of the mice were analyzed to determine the impact of treatment on survival duration, the time elapsed from functional ALS symptom onset until death (Figure 3B). The “survival dates” were collected from 16 studies that treated the B6SJL SOD1-G93A ALS mice with antioxidants. In the present study, survival date was defined as the day that the mouse expired as a result of ALS disease progression. A Mann-Whitney U-Test identified a significant difference between the survival dates for the treatment mice compared to untreated control mice (*p* < 0.00001). This result provides statistically significant evidence that treating with antioxidants does prolong survival in the preclinical SOD1-G93A pathology.

#### Impact of treatment initiation start date

**Figure 4:**
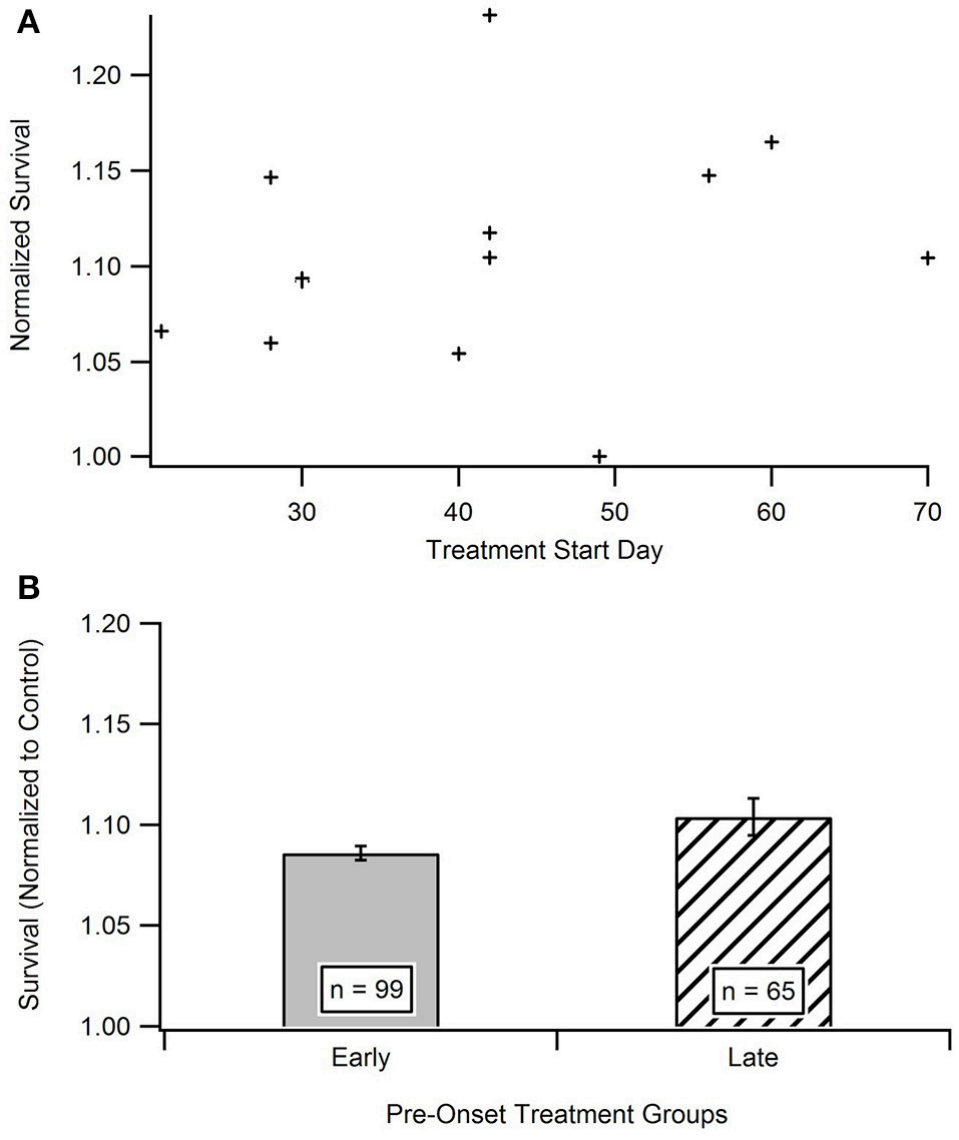
Comparison of early vs. late pre-onset treatment on animal survival. **(A)** Distribution of data points for time bin determination. The “early” pre-onset treatment group included mice that started treatments before day 42, and the “late” pre-onset treatment group consisted of mice that started treatments after day 42 (see Methods section Statistical Analysis). **(B)** Mean survival day for the early treatment group compared to late treatment group. A Mann Whitney U-test showed no statistically significant difference between the early and late pre-onset treatment groups.

Time is hypothesized to be a crucial factor when protecting against and treating ALS. In order to determine the existence of a relationship between treatment start date and survival date, the aggregated survival dates were separated into two groups based on when the treatments were initiated (Figure 4B). All treatments were started prior to functional symptom onset; as a reminder, the average functional symptom onset is 99 days for SOD1-G93A mice. The bi-modal time bins for the early pre-onset and late pre-onset treatment groups were determined by splitting the data set and removing the mid-section bin (see Methods section Statistical Analysis). Figure 4A illustrates a mild visual difference between the survival of mice treated earlier (average treatment initiation near 30-days) vs. later pre-onset (average treatment initiation near 60-days). A Mann-Whitney U-test showed that there is no significant difference) (*p* = 0.2864) in survival on the basis of pre-onset treatment start date. There was not enough studies that initiated treatment after ALS functional symptom onset to assess potential differences in results between pre-onset and post-onset treatment protocols (see Unraveling the Impact and Timing of Preclinical Antioxidant Therapy)

## Discussion

The following sub-sections discuss the widespread implications of antioxidant therapy for ALS and other neurological diseases impacted by oxidative stress while detangling some of the preclinical and clinical literature disparities. Furthermore, failed homeostatic regulation in the ALS oxidative stress response and potential methods to boost endogenous compensation are explored. For a short summary of major result highlights of the present study, please see the Conclusions.

### Unraveling the impact and timing of preclinical antioxidant therapy

The results of the presented *In vitro* aggregate analysis indicate that there is a direct correlation between oxidant concentration and cell death (Kriscenski-Perry et al., 2002; Petri et al., 2006a). However, it is striking to see a plateau in cell death after passing a certain value of free radical concentration, as was observed in this aggregate study. The observed plateau in cell death following prolonged intrinsic oxidative inducer exposure could be theorized as a “reset of death,” which might be attributed to a separate mechanism that is only activated under extreme conditions. Nonetheless, based solely on *In vitro* data, it would seem antioxidant treatments would have little to no effect on abating ALS disease progression after ROS concentration has exceeded the observed biological plateau threshold (Figure 1). That is, *In vitro* data emphasizes early intervention for maximal impact.

However, the aggregated *in vivo* data do not illustrate such a readily apparent ROS threshold that would dictate that antioxidant treatment must occur extremely early in the ALS disease course in order to have substantial impact. Antioxidants slowed *in vivo* post-onset progression, especially functional deficits measured via rotarod performance, across all disease stages. Interestingly, when antioxidant treatment was administered prior to onset, there was only a small, insignificant impact on delaying symptom onset (Figures 3A, 4). However, antioxidant treatments did reveal significant impacts on rotarod performance and survival (Figures 3B,C). Survival increased by about 11.2% in the treated mice, but the increases in muscle function (59.6%) were much more profound. The increases in muscle function present an opportunity to enhance quality of life of current patients while we await a curative or survival-enhancing therapy.

Ironically, in contrast to the plateau seen in the *In vitro* analysis, there was no difference seen in mouse survival based on “early” or “late” pre-onset *in vivo* treatment initiation (Figure 4). A more clinically relevant assessment would be to compare SOD1-G93A mice treated with antioxidants before onset vs. those initiating treatment after functional symptom onset. However, preclinical treatments almost exclusively treat mice prior to functional disease onset in order to better assess the impact of corresponding treatments on disease etiology. Nonetheless, given we cannot yet pre-identify what patients will get ALS or who is at high-risk, future preclinical post-onset ALS treatment exploration could have important clinical ramifications. Post-onset treatments that are successful in preclinical models will have a greater chance of translating into successful clinical therapies.

### Need for definitive metrics to compare oxidative stress etiology

Disparity between *In vitro* and *in vivo* experiments assessing oxidative stress cloud conclusions regarding the efficacy of antioxidant therapies. We believe the primary inconsistency exists because the antioxidants in *in vivo* assessment and the oxidants from the *In vitro* study target different aspects of oxidative stress. There are multiple factors that influence oxidative stress, and many more corresponding mechanisms that contribute to each factor. The antioxidants used in the *in vivo* study (Figures 3, 4) were largely anti-inflammatory—focused mostly on scavenging free radicals and removing other reactive oxidant species (ROS). Other anti-inflammatory drugs, such as NP001 (Miller et al., 2014) and carnosine (Caruso et al., 2017), have also proven to be helpful in treating ALS. Alternatively, the oxidants used in the *In vitro* study (Figure 1) were predominantly cytotoxic oxidants. To be able to consistently compare results in oxidative stress studies, it is necessary to define metrics that can fully encompass oxidative stress. Currently, there is no direct measure of “oxidative stress,” but only indirect measures of the mechanisms that contribute to oxidative stress. Future work is necessary to develop and standardize precise measures of oxidative stress in preclinical models that enable more direct etiological and therapeutic comparisons.

The present study focused predominantly on the SOD1-G93A preclinical model of ALS, the most common ALS model, in order to increase sample size and reduce heterogeneity. However, there are other preclinical models, including other types of ALS transgenic models (TDP-43, FUS, etc.) (Highley et al., 2014) that do not solely rely on the SOD mutation. Additionally, there are other experimental models that use cells derived from patients, such as patient-derived fibroblasts (Wray et al., 2012; Lenzi et al., 2015; Lim et al., 2016). Examination of overlap of in oxidative stress etiology between different disease models could be fruitful in determining common treatment targets for sporadic ALS, which makes up the preponderance of clinical cases. Moreover, any quantitative metric for oxidative stress needs to be translatable across different disease models in order for accurate and direct quantitative comparisons to be made.

### Translating preclinical antioxidant therapy success to the clinic

Interestingly, antioxidants were one of the first explored clinical therapies for ALS. The first clinical trial was with the antioxidant, Vitamin E, a peroxyl radical scavenger (Oliveira and Pereira, 2009). Later, the antioxidant melatonin, also a radical scavenger, was evaluated in both clinical and preclinical trials (Weishaupt et al., 2006). Clinical treatment effect sizes of oxidative stress therapies have been always <10%, which unfortunately, is less than the overall variance in disease durations of patients in the clinical trials. The large heterogeneity in patient survival durations, the primary metric used to determine therapy efficacy, is a key contributor to the lack of identified clinical therapies (Beghi, 2011). We contend clinical heterogeneity is directly responsible for disappointing clinical performance of antioxidants cited by a clinical retrospective study (Kamat et al., 2008). Simply put, the effect sizes of any statistically significant beneficial ALS therapy must be very large in order to outweigh the outcome metric and overall patient population variance.

In general, clinical trials using antioxidant treatments have shown lesser benefit compared to analogous preclinical animal studies (Kamat et al., 2008). Clearly ALS transgenic animal models do not have the same degree of population heterogeneity compared to predominantly sporadic clinical ALS patient populations. Moreover, *in vivo* preclinical treatments are administered prior to disease onset or minimally very early after mouse visible symptom onset. However, clinical ALS treatment typically begins much later in the disease course, largely because ALS is a diagnosis of exclusion (Kong et al., 2012)—meaning diagnosis is often delayed due to lack of a specific and sensitive ALS diagnostic test. Thus, the effect sizes of “positive” clinical trials must be comparatively much larger than their preclinical trial counterparts in order to overcome both the population heterogeneity and the later treatment initiation.

In summary, the present study illustrates that while antioxidants alone will likely not cure or stop ALS, they could be a key component as part of a multi-factorial combination therapy targeting this complex disease etiology (Mitchell and Lee, 2012b; Irvin et al., 2015; Kim et al., 2016). Moreover, antioxidants, especially those in the form of vitamins or supplements, which have relatively low side effects and risk profiles, could prolong muscle function and corresponding quality of life in ALS patients when initiated early in the disease course. Other clinical treatment possibilities include ways to modulate endogenous antioxidants and heat shock proteins, which in ALS, show an inadequate compensatory response (see Endogenous Antioxidant Therapy).

Finally, previous clinical trials with antioxidants only focused on survival benefit rather than quality of life or functional metrics, such as slowing muscular decline. The positive ramifications of low-risk antioxidants (such as those in vitamins, like Vitamin E or C) on ALS patient quality of life warrant further consideration of antioxidants as prophylactic and interventionist therapies for neurodegenerative disease. More work is necessary to characterize antioxidants in a clinical population, including developing ways to safely increase bioavailability through nutritional supplements or other delivery methods. A full assessment of antioxidant dosing, local uptake in the nervous system, and possible side effect profiles with increased bioavailability, is also needed.

### Innate homeostatic compensatory regulation fails in ALS

As shown in Figure 2, spinal HSP levels were significantly higher than that of muscles, which is consistent with prior work (Wei et al., 2013). This finding is explained by the higher activation threshold for the heat-shock response in motor neurons (Batulan et al., 2005). Having a higher HSP activation threshold means that muscle cells are more vulnerable to a hypothesized key factor of cell death due to protein misfolding, potentially explaining the earlier degeneration of muscle cells than spinal cells. The presented results support a possible retrograde mechanism proposed by the “dying-back” theory, which hypothesizes that ALS begins in the muscle cells or neuromuscular junctions (NMJ), and that degeneration of spinal cord motor neurons may occur because of the loss of muscle cells and degeneration of NMJ (Kiernan et al., 2011; Wei et al., 2013). It is likely that a multifactorial disease like ALS has been more than one etiology; as such, in some cases degeneration could initiate in the spine whereas in other cases perhaps at the muscle or NMJ.

Irrespective of the initiating site, it is clear that compensation for oxidative stress in ALS is insufficient. The homeostatic instability theory has been postulated to explain mathematical instability seen in an interactive dynamic, computational ALS model (Mitchell and Lee, 2012b), oscillations seen in preclinical experimental data compiled from the oxidative stress-respiration-apoptosis triad (Irvin et al., 2015), dynamic instability of type I and type II cytokines in ALS-induced inflammation in SOD1-G93A mice (Jeyachandran et al., 2015), and for the overzealous regulation thought to be tied to the lower prevalence of antecedent disease in ALS patients (Mitchell et al., 2015b; Hollinger et al., 2016). Thus, the present results of this study, and namely the insufficient HSP response (Figure 2), contribute further evidence that the etiology of ALS is propagated by system-wide homeostatic instability that can be initiated by multiple factors, especially in long motoneurons, which are known to be more vulnerable to instability than other neuron types (Kim et al., 2016).

### Endogenous antioxidant therapy

As discussed in section Innate Homeostatic Compensatory Regulation Fails in ALS, innate homeostasis, including responses to oxidative stress appear to fail in ALS (Mitchell and Lee, 2012b; Irvin et al., 2015). Beyond the innate heat shock proteins (HSPs), the body has other endogenous antioxidant mechanisms, such as glutathione or catalase (Kamat et al., 2008), which were not specifically examined in the present study. However, a previous preclinical ALS meta-analysis with glutathione (Irvin et al., 2015) showed its compensatory response to ALS oxidative stress was insufficient in ALS mice. While a small percentage of articles included in the present study mentioned glutathione, not enough additional preclinical articles specifically assessing glutathione were available to expand beyond the results of prior work (Irvin et al., 2015). Thus, the present work focused on innate preclinical HSP response and medicinal antioxidant therapy. Nonetheless, naturally occurring antioxidants and HSPs warrant further clinical research, including methods to modulate natural production or to supplement through exogenous administration of endogenous species. Of note is that the exogenous administration of supplemental HSP70 has been shown to be beneficial to the lifespan of SOD1-G93A mice (Gifondorwa et al., 2007).

### Oxidative stress as a common denominator in neurological disease

Oxidative stress impacts proteins and other genes within a continuum of neurological diseases that include ALS, Alzheimer's Disease (Foley et al., 2015; Huber et al., 2018), Parkinson's, Pick's disease, and others which have all shown to have many overlapping clinical biomarkers (Coan and Mitchell, 2015). One example of a shared antioxidant treatment is N-acetyl cysteine, which is noticeably effective at improving mitochondrial function (Díaz-Hung and González Fraguela, 2014). The aforementioned continuum of neurological diseases, as well as secondary post-traumatic brain and spinal cord injury (Mitchell and Lee, 2008), show apparent cellular metabolic dysfunction and reduced cellular regeneration due to the damaging effects of ROS. The physiological responses to ROS, antioxidants, and HSPs are relevant to all neuropathology but particularly those with identified mitochondrial dysfunction, protein misfolding, and inappropriate necro-apoptotic cell death responses (Andersen, 2004). More research is needed to examine common regulatory pathways in oxidative stress and redox homeostasis, which could be driving homeostatic instability and neurodegeneration in multiple types of neuropathology.

## Conclusions

Aggregate results of this metadata analysis indicate oxidative stress dramatically increases early in the ALS pathophysiology, well before symptom onset in SOD1-G93A transgenic ALS mice. Innate protective HSP proteins are qualitatively increased in an attempt to counter, but are unable to adequately compensate. Antioxidant treatments may not be able to stop ALS, as indicated by the negligible impact of pre-onset antioxidant therapy in delaying ALS onset. However, preclinical antioxidant therapy significantly extends survival by an average of 11.2% and significantly prolongs muscle function by an average of 59.6%. Thus, particularly given their large effect size on muscle function, antioxidants could have major implications for increasing clinical ALS patient quality of life.

## Author contributions

LB: data collection, statistical analysis of *in vivo* data, results interpretation, drafting of initial manuscript. KB: framing of study concept, data collection, statistical analysis of HSP data, drafting of initial manuscript. PM: data collection, statistical analysis of *In vitro* data, drafting of initial manuscript. KS: data collection, analysis of *in vivo* data, review of critical content. HS: data collection, analysis of *In vitro* data, review of critical content. CM: framing of study, project oversight, results interpretation, drafting of final manuscript, review of critical content.

### Conflict of interest statement

The authors declare that the research was conducted in the absence of any commercial or financial relationships that could be construed as a potential conflict of interest.
